# Appendectomy during the third trimester of pregnancy in a 27-year old patient: case report of a "near miss" complication

**DOI:** 10.1186/1754-9493-5-11

**Published:** 2011-05-17

**Authors:** Thomas Holzer, Gianmaria Pellegrinelli, Philippe Morel, Christian Toso

**Affiliations:** 1 Abdominal and Transplant Surgery, University Hospitals of Geneva, Faculty of Medicine, University of Geneva, Geneva, Switzerland

## Abstract

The management of acute appendicitis during pregnancy is not fully established, especially regarding the choice between open and laparoscopic surgery during the third trimester. We report herein the case of a major uterine variecele hemorrhage during a laparoscopic appendectomy in a 27-year old pregnant patient at 33 weeks of amenorrhea. After conversion to a Pfannenstiel incision, the baby was delivered, the bleeding stopped and the appendectomy completed. While both mother and child fully recovered, this «near miss» complication underlines the challenges linked to the management of acute appendicitis during pregnancy. Based on a literature review, we propose an algorithm favoring the laparoscopic approach during the first and second trimesters, and the open approach during the third trimester (especially after the 26th week of amenorrhea). In case of unclear pre-operative diagnosis, a laparoscopy should be conducted even during the third trimester with a Mc Burney conversion when the diagnosis of appendicitis is confirmed.

## Background

Appendicitis is the most common cause of non-obstetrical operation. Its incidence is approximately one per 1500 patients, which is similar to the one in the non-pregnant population.

The diagnosis of acute appendicitis is more challenging during pregnancy [1]. Up to 23% of appendectomies performed during pregnancy show normal appendices (versus 18% in non-pregnant patients, p < 0.05) [2]. This observation is mainly related to underlying physiological and anatomical changes leading to atypical pain, both in intensity and location [3-6]. In addition, a physiological leucocytosis is present during pregnancy and urine analysis often shows the presence of red blood cells or positive cultures, which can be misleading [4].

Multiple investigations and/or false diagnoses can delay surgery and increase the risk of appendix perforation, which is associated with higher rates of maternal morbidity (52% versus 17% non-perforated appendicitis) and foetal mortality (24% versus 7%) [4]. Conversely, performing an appendectomy for a false diagnosis of appendicitis is associated to at least similar rates of foetal loss and preterm delivery than regular appendectomies. Such complications may be related to the surgery itself and/or to the misdiagnosed disease [2,7,8].

Once the decision to operate has been made, the operative technique, open or laparoscopic surgery, must be decided. The laparoscopy is most often recommended during the first two trimesters, as alternative diagnoses can be evaluated in case of normal appendix [9-14]. During the third trimester, guidelines are less clear. Many speak for the open approach, especially after the 26^th^-28^th ^week of amenorrhea, but an increasing number of publications now report series of successful laparoscopic appendectomies during the third trimester [7,9,10,12-14].

The present case illustrates the risks and limitations of the laparoscopic approach during the third trimester of pregnancy and an algorithm based on the current literature is proposed for the management of appendicitis during pregnancy.

## Case report

A healthy 27-year-old pregnant woman presented to our emergency department at 33 weeks of amenorrhea with worsening abdominal pain. She demonstrated right iliac fossa tenderness. Blood tests showed transient leucocytosis at 11.5 g/l (norm: 4-11 g/l) and C - reactive protein of 68 mg/l (norm: 0-20 mg/l). The abdominal ultrasonography demonstrated a thin appendix with neighbouring infiltration and free fluid, compatible with appendicitis.

While the patient was on the list for an emergency appendectomy, she described a migration of the pain to the left iliac fossa, with a complete disappearance of the right sided symptoms. The on-call gynaecologist was consulted and premature labour was suspected. The patient was transferred to the obstetrical ward for tocolysis and assessment under epidural anaesthesia. With the exclusion of a spontaneous labour, the absence of sign of chorioamnionitis and the observation of a healthy foetus, the abdominal pain remained unexplained. After discussion with the gynaecologist, a laparoscopic exploration was undertaken with the aims to obtain a final diagnosis and to perform an appendectomy (if appropriate).

After the introduction of a 12 mm supra-umbilical trocar, advanced phlegmonous appendicitis with turbid free fluid was found. A 5 mm trocar was inserted in the right hypochondrium and a 12 mm trocar in the right flank. The working space was very narrow because of the gravid uterus and the dissection of the appendix was difficult due to multiple adhesions between the uterus, the bladder and the caecum.

After the appendix had been fully dissected, profuse bleeding was observed from the right parieto-colic gutter. Being unable to localize the precise source of bleeding, a Pfannenstiel incision was performed and a ruptured uterine varix was identified close to the right round ligament. The bleeding had potentially been induced by the friction of the right hand instrument against the uterus. Four sutures with adjunction of haemostatic matrix (Floseal, Baxter, Volketswil) were used without success. Although the patient remained hemodynamically stable, her haematocrit fell to 12%. The uterus was packed and an emergency C-section performed. After delivery, the uterus could be better mobilized, the bleeding was stopped with sutures and the appendectomy completed. The total blood loss was of approximately four liters. The patient received a total of seven red blood cell units, three units of fresh frozen plasma and one gram of fibrinogen during surgery. No transfusion was further required. The evolution of the haematocrit, thrombocyte count and coagulation tests has been summarized in table 1. The mother and new-born were discharged from hospital without further complication and are currently healthy 6 months after delivery.

**Table 1:** Evolution of hemoglobin, hematocrit, thrombocytes and coagulation with time.

	normal values	00:30 (preOP)	19:30 (perOP)	20:09 (perOP)	20:30 (perOP)	23:45 (J0 postOP)	03:30 (J1 postOP)	12:00 (J1 postOP)	06:00 (J2 postOP)
**Hemoglobin (g/L)**	**120-160**	105	92	43	71	133	*N/A*	*N/A*	116

**Haematocrit (%)**	**37-47**	30.8	27	12	20.7	39.2	*N/A*	*N/A*	32.1

**Thrombocytes (G/L)**	**150-300**	189	*N/A*	*N/A*	*N/A*	116	*N/A*	*N/A*	99

**Prothrombin time (%)**	**>70**	>100	*N/A*	*N/A*	86	*N/A*	>100	>100	*N/A*

**International normalised ratio**		1	*N/A*	*N/A*	1.06	*N/A*	1	1	*N/A*

**Partial thromboplastin time (sec)**	**26-37**	27.4	*N/A*	*N/A*	35	*N/A*	23.5	34.9	*N/A*

**fibrinogen (g/L)**	**1.5-3.5**	5	*N/A*	*N/A*	1.9	*N/A*	1.7	2.6	*N/A*

## Discussion

While some studies have demonstrated that the laparoscopic approach is feasible until 32-34 weeks of amenorrhea, conversion rates are higher and operative times longer [9,12-14]. This approach should, therefore, not be favoured during the third trimester. This said, we would still perform a laparoscopy during the third trimester in case of unclear pre-operative diagnosis. Once the intra-operative diagnosis of appendicitis has been made, we would favour a Mc Burney conversion (laparoscopy will guide the location of the open incision). We acknowledge however the difficulty of this decision, balancing a well progressing laparoscopic appendectomy, the potential complications to come and the safer profile of the open procedure.

Several key points may be helpful when the laparoscopic approach is chosen during pregnancy (whatever the trimester). They are based on the guidelines of the Society of American Gastrointestinal and Endoscopic Surgeons (SAGES): [15]

• An obstetrical consultation with foetal heart monitoring is recommended pre- and post-operatively.

• The patient should be positioned in the left lateral decubitus position to minimize the compression of the vena cava by the gravid uterus.

• CO^2 ^pressure should not be used higher than 10-15 mmHg. Ideally, the CO^2 ^pressure should be lower than 12 mmHg in order to minimize foetal acidosis and improve maternal ventilation. This should however be achieved with an appropriate working space.

• Maternal CO2 should be monitored by capnography.

• Trocar placement should be planned according to the location of the gravid uterus.

• Intra- and postoperative leg compression devices should be used to minimize the risk of thrombo-embolic events.

• There is no data regarding the use of unfractionated or low-molecular weight heparin during pregnancy but heparin has proven to be safe for prophylactic use.

• Tocolysis should not be used prophylactically but only in the presence of signs of preterm labor.

In case of major bleeding, an aggressive management should be performed, similar to those used in case of major bleeding after trauma [16].

**Figure 1:**
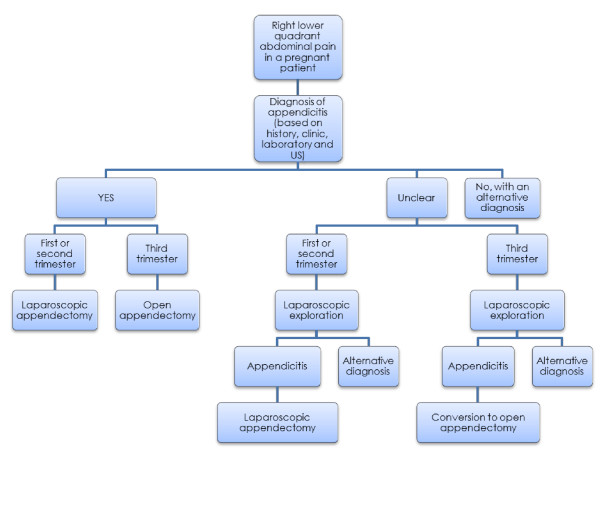
**Proposed management algorithm in case of right lower quadrant abdominal pain in a pregnant patient**.

Based on these observations, we propose a simple algorithm to improve the management of pregnant patients with acute right lower quadrant abdominal pain. (Figure 1) The open approach should be favoured in case of appendicitis during the third trimester of pregnancy.

## Conclusions

While more difficult in a pregnant woman, the diagnosis of acute appendicitis should be as prompt as possible in order to minimize maternal morbidity and foetal mortality. When the diagnosis is not clear, we recommend performing a laparoscopic exploration. During the third trimester we recommend a conversion to an open appendectomy once the intra-operative diagnosis of appendicitis has been made.

## Consent

Written informed consent was obtained from the patient for publication of this case report. A copy of the written consent is available for review by the Editor-in-Chief of this journal.

## Competing interests

The authors declare that they have no competing interests.

## Authors' contributions

TH and CT have taken part to the surgery and have written the case report. PG has taken part to the surgery and reviewed the case report. PM has reviewed the case report. All authors read and approved the final manuscript.

## References

[B1] Ueberrueck T Koch A Meyer L Hinkel M Gastinger I Ninety-four Appendectomies for Suspected Acute Appendicitis during Pregnancy World Journal of Surgery 2004 28 5 508 511 10.1007/s00268-004-7157-2 15085399

[B2] McGory M Zingmond D Tillou A Hiatt J Ko C Cryer H Negative Appendectomy in Pregnant Women Is Associated with a Substantial Risk of Fetal Loss J Am Coll Surg 2007 205 4 534 540 10.1016/j.jamcollsurg.2007.05.025 17903726

[B3] Horowitz M Gomez G Acute appendicitis during pregnancy. Diagnosis and Management Arch Surg 1985 120 12 1362 1367 4062542 10.1001/archsurg.1985.01390360028007

[B4] Yilmaz H Akgun Y Bac B Celik Y Acute appendicitis in pregnancy -- risk factors associated with principal outcomes: A case control study Int J Surg 2007 5 3 192 197 10.1016/j.ijsu.2006.05.005 17509502

[B5] Pastore P Loomis D Sauret J Apendicitis in Pregnancy JABFM 2006 19 6 621 626 10.3122/jabfm.19.6.621 17090795

[B6] AL-Mulhim A Acute appendicitis in pregnancy. A review of 52 cases Int Surg 1996 81 3 295 297 9028994

[B7] Machado N Grant C Laparoscopic appendicectomy in all trimesters of pregnancy JSLS 2009 24 2 383 389 PMC3015967 19793481

[B8] Walsh C Tang T Walsh S Laparoscopic versus open appendicectomy in pregnancy: A systematic review International Journal of Surgery 2008 6 4 339 344 10.1016/j.ijsu.2008.01.006 18342590

[B9] Halkic N Tempia-Caliera AA Ksontini R Suter M Delaloye J-F Vuilleumier H Laparoscopic management of appendicitis and symptomatic cholelithiasis during pregnancy Langenbeck's Archives of Surgery 2006 391 5 467 471 10.1007/s00423-006-0069-x 16909295

[B10] Perrot Md Jenny A Morales M Kohlik M Morel P Laparosocpic appendectomy during pregnancy Surg Laparosc Endosc Percutan Tech 2000 10 6 368 371 11147911

[B11] Rollins MD Chan KJ Price RR Laparoscopy for appendicitis and cholelithiasis during pregnancy: a new standard of care Surgical Endoscopy 2004 18 2 237 241 10.1007/s00464-003-8811-8 14691706

[B12] Lemieux P Rheaume P Levesque I Bujold E Brochu G Laparoscopic appendectomy in pregnant patients: a review of 45 cases Surgical Endoscopy 2008 23 8 1701 1705 19057956 10.1007/s00464-008-0201-9

[B13] Upadhyay A Stanten S Kazantsev G Horoupian R Stanten A Laparoscopic management of a nonobstetric emergency in the third trimester of pregnancy Surgical Endoscopy 2007 21 8 1344 1348 10.1007/s00464-006-9104-9 17285387

[B14] Sadot E Telem DA Arora M Butala P Nguyen SQ Divino CM Laparoscopy: a safe approach to appendicitis during pregnancy Surgical Endoscopy 2009 24 2 383 389 19551438 10.1007/s00464-009-0571-7

[B15] Guidelines for diagnosis, treatment, and use of laparoscopy for surgical problems during pregnancy Surgical Endoscopy 2008 22 4 849 861 18288533 10.1007/s00464-008-9758-6

[B16] Rossaint R Bouillon B Cerny V Coats T Duranteau J Management of bleeding following major trauma: an updated european guideline Critical care 2010 10.1186/cc8943 PMC2887168 20370902

